# Expanding the coverage of regulons from high-confidence prior knowledge for accurate estimation of transcription factor activities

**DOI:** 10.1093/nar/gkad841

**Published:** 2023-10-16

**Authors:** Sophia Müller-Dott, Eirini Tsirvouli, Miguel Vazquez, Ricardo O Ramirez Flores, Pau Badia-i-Mompel, Robin Fallegger, Dénes Türei, Astrid Lægreid, Julio Saez-Rodriguez

**Affiliations:** Heidelberg University, Faculty of Medicine, and Heidelberg University Hospital, Institute for Computational Biomedicine, Bioquant, Heidelberg, Germany; Department of Clinical and Molecular Medicine, Norwegian University of Science and Technology, Trondheim, Norway; Department of Biology, Norwegian University of Science and Technology, Trondheim, Norway; Barcelona Supercomputing Center, Barcelona, Spain; Heidelberg University, Faculty of Medicine, and Heidelberg University Hospital, Institute for Computational Biomedicine, Bioquant, Heidelberg, Germany; Heidelberg University, Faculty of Medicine, and Heidelberg University Hospital, Institute for Computational Biomedicine, Bioquant, Heidelberg, Germany; Heidelberg University, Faculty of Medicine, and Heidelberg University Hospital, Institute for Computational Biomedicine, Bioquant, Heidelberg, Germany; Heidelberg University, Faculty of Medicine, and Heidelberg University Hospital, Institute for Computational Biomedicine, Bioquant, Heidelberg, Germany; Department of Clinical and Molecular Medicine, Norwegian University of Science and Technology, Trondheim, Norway; Heidelberg University, Faculty of Medicine, and Heidelberg University Hospital, Institute for Computational Biomedicine, Bioquant, Heidelberg, Germany

## Abstract

Gene regulation plays a critical role in the cellular processes that underlie human health and disease. The regulatory relationship between transcription factors (TFs), key regulators of gene expression, and their target genes, the so called TF regulons, can be coupled with computational algorithms to estimate the activity of TFs. However, to interpret these findings accurately, regulons of high reliability and coverage are needed. In this study, we present and evaluate a collection of regulons created using the CollecTRI meta-resource containing signed TF–gene interactions for 1186 TFs. In this context, we introduce a workflow to integrate information from multiple resources and assign the sign of regulation to TF–gene interactions that could be applied to other comprehensive knowledge bases. We find that the signed CollecTRI-derived regulons outperform other public collections of regulatory interactions in accurately inferring changes in TF activities in perturbation experiments. Furthermore, we showcase the value of the regulons by examining TF activity profiles in three different cancer types and exploring TF activities at the level of single-cells. Overall, the CollecTRI-derived TF regulons enable the accurate and comprehensive estimation of TF activities and thereby help to interpret transcriptomics data.

## Graphical Abstract

**Graphical Abstract:**
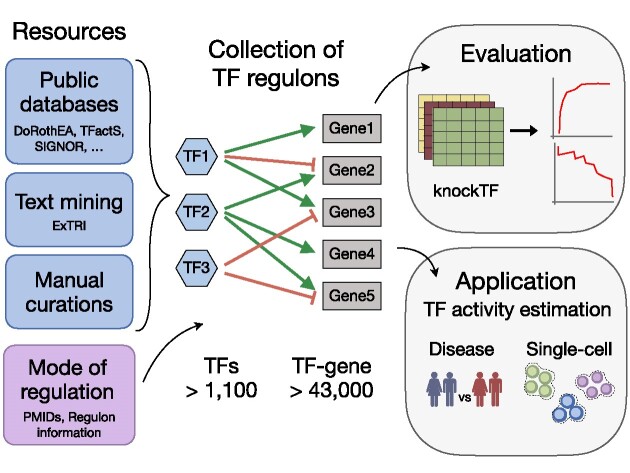

## Introduction

The regulation of gene transcription plays a fundamental role in development, cell differentiation, tissue homeostasis, and various physiological processes and is crucial for the coordinated response of cells to both internal and external signals. As such, its dysregulation can contribute to the development of numerous diseases, including cancer, autoimmunity, neurological disorders, developmental syndromes, diabetes, or cardiovascular disease (1). In particular, deregulated activity of transcription factors (TFs)—key regulators of transcription - has been implicated in the development of cancer and can generally alter the core autoregulatory circuitry of a cell (1–3). Transcription factors bind to specific regions of the DNA and together with cofactors and other proteins influence the transcriptional rate of a specific set of target genes (TGs) (4) collectively known as the TF’s regulon. The combined interactions of all TFs to their target genes are often referred to as a gene regulatory network (GRN), a simplified representation of the underlying regulatory circuits (5). Coupling GRNs with activity inference algorithms (6) can facilitate the interpretation of transcriptomics data and provide a more effective means of understanding the underlying regulatory mechanisms in the system of interest. TF activity estimation has been used to better understand diseases, for example breast cancer (7), human myocardial infarction (8) and oligodendroglioma (9), drug response (2,10,11), immunotherapy (12), aging (13) and development (14). Since the choice of TF-regulons can substantially affect the results (15), it is important to use TF-regulons of high quality, minimizing false-positive interactions, while having the highest coverage possible to not miss potentially relevant TFs.

Various methods are available for identifying TF regulons, both on a small and large scale. Experimentally, high-resolution identification of TF binding sites *in vivo* can be achieved using chromatin immunoprecipitation followed by high-throughput DNA sequencing (ChIP-seq) (16) and DNase I hypersensitivity coupled with DNA sequencing (DNase-seq) (17). Despite identifying TF–DNA binding events in their native environment, binding events might not correspond to actual changes in the expression of the target gene, and do not take into account other cofactors that bind indirectly to the target genes (18). *In silico*, the prediction of TF–gene interactions can be done using the genomic sequence recognised by each TF, also known as binding motifs (19,20). Such methods involve probing the entire genome for regions that contain these binding motifs to identify potential target genes. However, this approach is limited to TFs with known binding motifs and does not account for context-specific interactions, where regulatory interactions take place only in a specific cell type or condition. Furthermore, GRNs can be inferred in a data-driven manner, as in co-expression analysis where the correlation between the expression patterns of a TF and its potential target genes is investigated (21). Lastly, manual curation of TF–gene interaction from the literature is another common strategy. Such curation efforts are usually incorporated in databases such as IntAct (22), SIGNOR (23) and TRRUST (24). While being very attractive for their high quality, manual curations are hard to come by since curation is a generally cumbersome task, and curated databases rarely overlap due to the different curation standards and protocols (15,25). For that, curation efforts can be significantly enhanced with the aid of text-mining for the identification of TF–gene interactions, as previously shown (26).

Despite the availability of the afore-described methods, the lack of a general consensus on the inference of TF–gene interactions remains a challenge as each approach has its own strengths and limitations. A few frameworks to create TF regulons based on a combination of resources have been proposed. Such frameworks include DoRothEA (15), which combines TF–gene interactions identified by ChIP-seq experiments, inferred interactions by gene expression and TF binding motifs and manual curation, and ChEA3 (27), which primarily contains co-expression and ChIP-seq-inferred interactions. Other examples include Pathway Commons, which is a resource that integrates various types of interactions (i.e. biochemical, complex binding and physical interactions between proteins, RNA, DNA and small molecules) (28) and RegNetwork, which compiles experimentally observed or motif-based predicted interactions among TFs, microRNAs and target genes (29). However, such meta-resources can include a high number of false interactions due to the use of high false-positive generating methods (i.e. co-expression and co-occurrence) (30), and, with the exception of DoRothEA, they do not include the information about the sign of interactions (i.e. activating or inhibiting interactions).

In this study, we introduce a set of TF regulons created using information on TF–gene interactions from the CollecTRI (Collection of Transcription Regulation Interactions) meta-resource (26) which integrates multiple sources of interaction data, including public databases, text mining, and manual curation. The CollecTRI-derived regulons represent 43175 signed TF–gene interactions for 1186 TFs. Additionally, we propose a workflow for defining the sign of interactions (activating or repressing) based on (i) information about the sign curated in the resources compiled in CollecTRI and on (ii) regulon properties to assign a predominantly activating or repressing role to TFs. The proposed workflow can also be applied to other comprehensive knowledge bases. We benchmarked the performance of CollecTRI-derived regulons against TF regulons from four other meta-resources: DoRothEA, ChEA3, RegNetwork and Pathway Commons. The CollecTRI-derived regulons outperformed the other networks by accurately inferring changes in TF activities in TF perturbation experiments collected in the KnockTF data (31). Lastly, we demonstrated the value of the CollecTRI-derived regulons through two different case studies. We estimated the differential activities of TFs in three cancer types using public data from the Clinical Proteomic Tumor Analysis Consortium (CPTAC) and were able to identify TFs with known roles in cancer biology and the studied cancer types. Furthermore, we used the CollecTRI-derived regulons to estimate TF activities on a single-cell dataset of peripheral blood mononuclear cells (PBMCs) and identified cell-type marker TFs. In summary, we provide a new high-confidence, high-coverage collection of TF-regulons that we make freely available via the OmniPath (https://omnipathdb.org/) (32), DoRothEA (https://saezlab.github.io/dorothea/) (15) and decoupler (https://saezlab.github.io/decoupleR/) (33) packages.

## Materials and methods

### TF–gene data sources

The CollecTRI source data were introduced in (26) and have since been updated by gathering more recent data from SIGNOR and GO, and by adding three new resources: DoRothEA A (15), Pavlidis 2021 (34) and the NTNU Curated subset of ExTRI (Lægreid, in preparation). Pavlidis consists of information extracted from the supplemental materials of the publication. The code that implements the gathering and merging of the data is available within the ExTRI Rbbt workflow (https://github.com/Rbbt-Workflows/ExTRI). Each data source is processed to a common format with transcription factors and target genes expressed as human gene symbols. For databases that list genes and proteins of different organisms, the UniProt protein to protein identifier equivalences from the Protein Information Resource (https://proteininformationresource.org/) were used to identify the proper human protein, which was then translated to its gene symbol (35). Each resource was processed to update all gene symbols to the most recent version (gene set in Ensembl release 109 from February 2023). When merging databases, entries for AP1 and NFKB complex members are allowed to match the complex names so that their information is merged across them. The result of this process is a table where each TRI (TF–gene pair) is listed with its databases of origin, along with information from those databases, such as mode of regulation, when available, and literature references provided as PubMed ID (PMID).

### Filtering TF–gene interactions

CollecTRI, as well as all other regulon collections (RegNetwork, ChEA3, Pathway Commons, DoRothEA), were filtered to contain only transcription factor (TF)–gene interactions from TFs classified as DNA-binding (dbTFs), co-regulatory (coTFs) or general initiation (GTFs). dbTFs were downloaded from Lambert *et al.* (https://ars.els-cdn.com/content/image/1-s2.0-S0092867418301065-mmc2.xlsx), Lovering *et al.* (https://ars.els-cdn.com/content/image/1-s2.0-S1874939921000833-mmc1.xlsx) and TFclass (http://tfclass.bioinf.med.uni-goettingen.de/suppl/tfclass.ttl.gz). Additionally, we retained all proteins annotated in Gene Ontology (GO) (36) with either the specific term or any child term of *DNA-binding transcription factor activity* (GO:0003700) for dbTFs, *transcription coregulator activity* (GO:0003712) for coTFs or *general transcription initiation factor activity* (GO:0140223) for GTFs through QuickGO (37) on 2023-03-07.

### Assigning the TF–gene mode of regulation

For each TF–gene interaction from the CollecTRI meta-resource the PMIDs were aggregated across databases. Each PMID is considered evidence of whichever mode of regulation is specified in that database, if any. Database entries that are not supported by PMIDs are thus not considered when building the TF regulons. PMIDs can only count as evidence for a TF–gene interaction once, even if they were curated by several databases. Only in the infrequent case of the same PMID featured as supporting different modes of regulation in different databases, these were considered twice for determining the mode of regulation to use in the CollecTRI-derived regulons.

We then explored different strategies to assign a mode of regulation (activating or repressing) to each TF–target interaction based on multiple sources of information. Initially, we compared two approaches: assigning the mode of regulation per TF–target interaction based on the prevalence of PMIDs associated with a specific mode, versus assigning a mode of regulation for the entire regulon of a TF based on its general mode of regulation. To determine the general mode of regulation for a TF, we extracted regulatory information from GO terms (36) and Uniprot keywords (38), as well as structural information about the Krüppel associated box (KRAB) domain and the characterization and classification of effector domains of 594 human TFs provided by Soto *et al.* (39). More specifically we checked if the TF was annotated with either the specific term or any child term of *RNA polymerase II-specific DNA-binding transcription activator activity* (GO:0001228), *DNA-binding transcription repressor activity* (GO:0001217), *transcription coactivator activity* (GO:0003713), *transcription corepressor activity* (GO:0003714), *positive regulation of transcription by RNA polymerase II* (GO:0045944) or *negative regulation of transcription by RNA polymerase II* (GO:0000122). From the UniProt keywords we extracted the information on the TF role based on the UniProtKB keywords Activator (KW-0010) and Repressor (KW-0678). KRAB-proteins were assigned a repressive mode of regulation and identified by their InterPro-membership (40) in the IPRO3651_superfamily, while excluding members of the IPRO03655_ancient KRAB family, as the ancient KRAB proteins are known to act both as repressors and activators. If all sources—GO term classification, UniProt keywords, KRAB domain presence and effector domain information—agreed on the regulatory effect of a TF, we assigned the mode of regulation to all target genes accordingly. Overall, we identified 348 TFs classified as general activators and 232 classified as general repressors leading to a total of 10 313 and 3191 TF–gene links where we assigned an activating and repressing mode of regulation, respectively, using this strategy. In comparison, we assigned an activating and repressing mode of regulation to 13 847 and 5694 TF–gene links, respectively, based on the prevalence of PMIDs. Since both approaches resulted in a majority of interactions being assigned a positive mode of regulation (PMIDs: 71%, TF classification: 76%), we also assigned an activating mode of regulation in cases where no information was available from either the PMIDs or the regulatory effect of a TF. We evaluated both approaches individually by comparing their performance in our benchmarking approach (See methods: Benchmark procedure) against the CollecTRI version, where the mode of regulation of all edges was considered to be activating. Additionally, we examined the effect of combining both approaches: First, we assigned a mode of regulation based on the PMIDs for TF–gene interactions, and where this information was not available, assigned a mode of regulation based on the general mode of regulation for the TF, defined by the GO terms and UniProt keywords. All remaining TF–gene interactions were again assigned an activating mode of regulation. Furthermore, we considered information from other interactions within a TF’s regulon to assign a likely activating or repressing mode of regulation to the TF. We analyzed all TF–gene interactions in the regulon with an assigned mode of regulation based on the PMIDs and classified the TF based on whether the majority of these interactions were linked to activation or repression. We then assigned the mode of regulation accordingly, resulting in an activating and repressing mode of regulation for 1750 and 154 TF–gene links, respectively and again tested the performance in our benchmark approach. Additionally, we evaluated the effect of adding the general mode of regulation for a TF as before, to all TF–gene interactions without any information from the PMIDs and the regulon classification,

Lastly, we systematically compared the effect of assigning an activating or repressing mode of regulation by default in cases where no information was available from any of the sources.

After evaluating the benchmarks, in the published CollecTRI-derived regulons we assigned the mode of regulation for each TF–gene interaction primarily based on the number of literature references and secondarily based on other TF–gene interactions in the regulon. TF–gene interactions without any information were assigned an activating mode of regulation.

### Implementation of the CollecTRI-derived regulons in OmniPath

After filtering the CollecTRI-derived regulons to include only TF–gene interactions from regulators classified as TFs (Methods: Filtering TF–gene interactions), and assigned the mode of regulation to each TF–gene link (Methods: Assigning the TF–gene mode of regulation), we incorporated the CollecTRI regulons into the OmniPath database (32), enabling convenient distribution and integration with other databases. We created methods in Pypath, the database builder of OmniPath, for processing CollecTRI. The database build process ensures that each gene is represented by its primary UniProt ID and primary HGNC symbol. It also translates the interactions to their mouse and rat counterparts by orthologous gene pairs. In OmniPath, all varieties of complexes ‘AP1’ and ‘NFKB’ are listed explicitly, according to Bejjani *et al.* (41) and Hoffmann *et al.* (42). For ‘AP1’ we did not include the extended definition described in Bejjani et al. but solely considered members from the Jun and Fos families in the dimer collection. Integrated into OmniPath, CollecTRI is distributed in the web service at https://omnipathdb.org/ along with other OmniPath datasets, enabling access by the OmniPath Python, R and CytoScape clients.

### RegNetwork, ChEA3, pathway commons and DoRothEA

RegNetwork (29) human regulons were downloaded from their website (https://regnetworkweb.org/download.jsp). The TF regulons from ARCHS4_Coexpression (ChEA3 ARCHS4), ENCODE_ChIP-seq (ChEA3 ENCODE), Enrichr_Queries (ChEA3 Enrichr), GTEx_Coexpression (ChEA3 GTEX), Literature_ChIP-seq (ChEA3 Literature) and ReMap_ChIP-seq (ChEA3 ReMap) were downloaded from their website (https://maayanlab.cloud/chea3/) (27). DoRothEA (15) was downloaded using the function get_dorothea from the *decoupler v2.4.0* bioconductor package (33) once filtered by its confidence levels A, B and C and once including all confidence levels (A, B, C, D). For each TF–gene interaction where the sign of regulation was not stated, an activating mode of regulation was assigned by default.

### Computing TF–gene weights

We employed two different tools, namely *MatrixRider* (43) and *FIMO* (44), to perform motif enrichment analysis and calculate binding weights for the TF–gene interactions in the CollecTRI-derived regulons. Specifically, we used the *Matrixrider v1.30.0* and *memes v1.6.0* bioconductor packages. Before running the methods, we extracted 1000 base pairs (bp) upstream and 100 bp downstream of the transcription start site of each gene (TSS), defining the promoter region, as well as 10 000 base pairs (bp) upstream and 100 bp downstream of the TSS, reflecting proximal regulatory regions using the *TSS.TxDb.Hsapiens.UCSC.hg38.knownGene v3.4.6* package (45). Human TF binding motifs were downloaded from *MotifDb v1.40.0* (46). TF–gene pairs for which either the promoter sequence or the TF binding motif were not available were removed from the network. For the remaining 40 440 TF–gene pairs the two different tools were used as follows. MatrixRider was used to calculate binding weights for each TF–gene interaction as described in their reference manual. TF binding motifs were provided as position frequency matrices, DNA sequences of the target genes as DNAString objects and a cutoff parameter of 0 were passed to the getSeqOccupancy function within the *Matrixrider v1.30.0* package. For FIMO, TF binding motifs and DNA sequences were passed to the runFimo function within the *memes v1.6.0* package and the highest score was kept as the binding weight. For both methods, calculated binding weights were shifted to positive values with a pseudo count of 1 and normalized. We used two different normalization strategies. First, we normalized the weights per TF, meaning that the weights for all targets of a specific TF were divided by the highest TF–gene binding weight of that TF. Secondly, we normalized the weights per gene, meaning that the weights for all TFs regulating a specific gene were divided by the highest TF–gene binding weight of that gene. The final weights were compared with each other using Pearson correlation. The benchmark procedure was then repeated for the weighted network, using the calculated weights from MatrixRider with a window frame of 1000 bp before normalization, and compared to the non-weighted CollecTRI regulons. Additionally, TF–gene links with binding weights in the lowest 10, 20 and 30% quantile were removed from the network and their performance evaluated in the benchmark.

### Benchmark data

Differentially expressed gene tables and meta data were downloaded from 907 manually curated RNA-seq and microarray experiments, collected in knockTF (31) (http://www.licpathway.net/KnockTF/download.php). These datasets include knockdown/knockout experiments across multiple tissues and cell types associated with 456 different disrupted TFs. Perturbation experiments with a perturbed TF’s log fold change greater than –1 were excluded from the final benchmark set, leading to 388 data sets covering 234 unique perturbed TFs. For each resource only perturbation experiments of TFs covered in that network were used for the benchmark (Table 1).

**Table 1.** Overview tested TFs per network

Network	Total number of TFs covered in benchmark
ChEA3 ARCHS4	156
ChEA3 ENCODE	46
ChEA3 Enrichr	146
ChEA3 GTEX	155
ChEA3 Literature	66
ChEA3 ReMap	101
CollecTRI	171
DoRothEA ABC	125
RegNetwork	158
Pathway Commons	121

### TF activity estimation

TF activities were estimated based on the log fold-changes of the direct target genes after perturbation. Each network was first filtered to keep only TF–gene interactions of genes measured in the experiment. We then selected TFs with at least five gene targets and estimated TF activities using the univariate linear model (ulm) method from the *decoupler v1.2.0* Python package (33).

### Benchmark procedure

The benchmark was performed using the benchmark function from the *decoupler v1.2.0* Python package (33). To globally evaluate collections of TF regulons, TF activity scores obtained as described above are first multiplied by the sign of the perturbation (knockout: negative, overexpression: positive) for each perturbation experiment. The activity scores matrix (rows: experiments, columns: TF activities) is then flattened across experiments into a single vector. The objective is to distinguish between perturbed TFs, the true positives, from all unperturbed ones, true negatives. Due to differences in class imbalance across networks, a downsampling strategy is employed within the benchmark. For each permutation, an equal number of positive and negative classes are randomly selected to calculate the area under the Receiver Operating Characteristic (AUROC) and Precision-Recall Curve (AUPRC) metrics. This process is repeated 1000 times per network, obtaining distributions of performance measurements.

The performance evaluation for specific TFs was performed only for TFs for which at least five experiments were available in the KnockTF dataset (after filtering out experiments with insufficient perturbation, Methods: Benchmark data). In this setting, the objective is to distinguish between perturbed experiments for each retained TF, the true positives, from all the unperturbed ones, true negatives. The same strategy as described above is used, but instead of flattening the activity scores matrix, only the vector of one TF is extracted for evaluating the performance. This was done separately for each of the TFs in Supplementary Figure S3.

### Evaluation of size bias

For the three top performing regulon collections, namely CollecTRI, DoRothEA ABC and RegNetwork, we used a two-sided *t*-test which was adjusted for multiple testing using Benjamini–Hochberg correction to compare if there was a difference in the number of targets for TFs that were part of the benchmark data set, compared to the TFs that were not. Pearson correlation coefficients were computed to assess the relationship between the number of targets and the absolute activity scores of TFs across all benchmark experiments included in the benchmark. We then summarized the correlation between the absolute scores and the number of targets across experiments with the mean correlation and compared it across networks.

### Case study 1: TF estimation in cancer tissues

We tested the network by estimating TF activities in three types of cancer: Uterine Corpus Endometrial Carcinoma (UCEC), Lung Adenocarcinoma (LUAD), and Clear Cell Renal Cell Carcinoma (CCRCC), retrieved from the third phase of the Clinical Proteomic Tumor Analysis Consortium (CPTAC). The data was provided by Gaytan et al. (in preparation) which was collected from NCI’s Genomic Data Commons (GDC) (47) using the GDC transfer tool. Raw count tables were subjected to VSN normalization, after filtering genes with a low number of counts (48). For each cancer type, we then performed differential expression analysis between tumor and normal tissue samples using the *limma* R package (49). The *t*-values were used to infer TF activity as described previously. A significance threshold of 0.05 was applied. Additionally, the correlation between TF activity and TF expression was calculated.

### Case study 2: TF estimation in single-cell RNA data

#### Single-cell RNA-seq processing

Single-cell RNA-seq data from peripheral blood mononuclear cells (PBMCs) was downloaded from 10x Genomics (https://cf.10xgenomics.com/samples/cell/pbmc3k/pbmc3k_filtered_gene_bc_matrices.tar.gz) and processed according to the standard preprocessing workflow as described in the Seurat (50) tutorial using *Seurat v4.3.0*: Cells with >5% mitochondrial counts or unique feature counts >2500 or <200 were filtered using the subset function. The feature expression measurements for each cell in the data was then normalized by the total expression, multiplied by a scaling factor of 10 000 and log-transformed with the NormalizeData function. The FindVariableFeatures function was utilized to calculate 2000 features with high cell-to-cell variations, employing ‘vst’ as the selection method. Next, a linear transformation was applied using the ScaleData function. On the scaled data, principal component analysis (PCA) was performed using the calculated variable features. For clustering, the cells were embedded in a K-nearest neighbor graph based on the euclidean distance in PCA space with edges drawn between cells with similar feature expression patterns using the FindNeighbors function. We next used the Louvain algorithm to iteratively group cells together using the FindClusters function with a resolution of 0.5. The clusters were visualized using uniform manifold approximation and projection (UMAP). Marker genes defining each cluster were identified via differential expression using the FindAllMarkers function. Only genes with a log fold change >0.25 and an expression observed in at least 25% of the cells in a cluster were considered to be markers. Clusters were annotated by mapping marker genes to canonical cell type markers taken from the Seurat tutorial.

#### TF activity estimation in PBMCs

For each cell in the PBMC dataset we employed the univariate linear model method implemented in *decoupleR v2.4.0* to estimate TF activities from the normalized counts using the CollecTRI-derived regulons. To identify TF markers for each cell type within the PBMC dataset, we applied the FindAllMarkers function using a Wilcoxon test from *Seurat v4.3.0* on the expression of all TFs and the inferred activity of all TFs. TFs with an adjusted p-value smaller than or equal to 0.05, a log fold change >0.5 and an expression or activity observed in at least 15% of the cells in a cell type were considered to be markers for that specific cell type. The total number of TF cell type markers based on expression and activity were then quantified.

To evaluate if the activities of transcription factors are better conserved than their expression within cell types, we assessed the potential of TF expression and activity to cluster cells from the same cell type together. For both the expression and the activity matrix we performed the following steps: we applied a linear transformation using the ScaleDate function from Seurat v4.3.0 and performed PCA using all TFs. We calculated the euclidean distance in the first 30 PCA dimensions and computed the silhouette width using the silhouette function from the cluster v2.1.4 R package (51). Silhouette widths of all cells were aggregated and the average silhouette width derived from TF activity and TF expression compared using a two-sided *t*-test.

## Results

### Sources of prior knowledge on TF–gene regulatory interactions

CollecTRI (Collection of Transcription Regulation Interactions) is a compilation of available transcription regulation information from databases integrated with information extracted from the text-mined resource ExTRI (26), which extracts sentences containing descriptions of transcription regulation events, known as TRIs (Transcription Regulation Interactions). Here, we used those resources after updating some of the databases and including three new ones (Table 2): Pavlidis (34), NTNU Curated (Lægreid, in prep) and DoRothEA A (15) (Supplementary Figure S1). NTNU Curated are a subset of ExTRI sentences manually curated for validity of TF–gene interaction and mode of regulation, and DoRothEA A contains the TF–gene interactions with the highest confidence level of the DoRothEA meta-resource (15), which is also evaluated in this publication separately. Note that DoRothEA compiles some of the same resources as CollecTRI; however, the overlap is taken into account for the creation of the CollecTRI-derived regulons as it is based on the number of unique PubMed IDs (PMIDs) supporting each annotation across resources.

**Table 2.** Resources of TF–gene interactions used in CollecTRI

Database	Content extracted for compilation	Reference
ExTRI	All (cross-species)	Vazquez, 2022 (26)
TFactS	All (human, mouse, rat)	Essaghir, 2010 (53)
HTRIdb	All (human)	Bovolenta, 2012 (54)
IntAct	Subset: protein-gene interactions (human, mouse, rat)	Kerrien, 2012 (55)
GOA (updated)	Subset: protein-gene regulatory interactions (human, mouse, rat)	Huntley, 2015 (56)
TRRUST	All (human, mouse)	Han, 2015; Han, 2018 (24,57)
SIGNOR (updated)	Subset: interactions labeled with interaction mechanism ‘transcriptional regulation’ (human, mouse, rat)	Perfetto, 2016 (23)
CytReg	All (human, mouse)	CarrascoPro, 2019 (58)
GEREDB	Subset: interactions with regulator TFClass TF (human)	Huang, 2019 (59)
Pavlidis (new)	All (human, mouse)	Pavlidis, 2021 (34)
DoRothEA A (new)	All (human)	Garcia-Alonso, 2019 (15)
NTNU Curation (new)	Subset of about 20K sentences manually curated from ExTRI	Lægreid, in prep.

The table indicates whether all interactions or subsets of them were included in CollecTRI.

From the resources, all instances of genes or proteins have been translated into human gene symbols, including mentions to rat or mouse entities which were translated with the help of orthology tables. We decided to also consider TF–gene interactions from mouse and rat, as it is a common practice in the field to assume that the TRIs translate across murine organisms and humans due to the high conservation of regulatory mechanisms across these organisms (52). Additionally, a large component of CollecTRI is extracted from text-mining, where it is often difficult to assign the correct species to information extracted from PubMed abstracts; in fact, this information may be missing entirely from abstracts.

Moreover, two TF dimers, AP1 and NFKB, were treated as transcription factors themselves in CollecTRI since they, in the literature, are very frequently mentioned only by their dimer name. When merging the CollecTRI resources, the information regarding the monomer AP1 or NFKB constituents (e.g. JUN- or FOS-family or NFKB1) was merged into information referring to the complex (i.e. AP1 for JUN and FOS) and vice versa, see details in (23).

### Construction of TF regulons from CollecTRI

**Figure 1.**
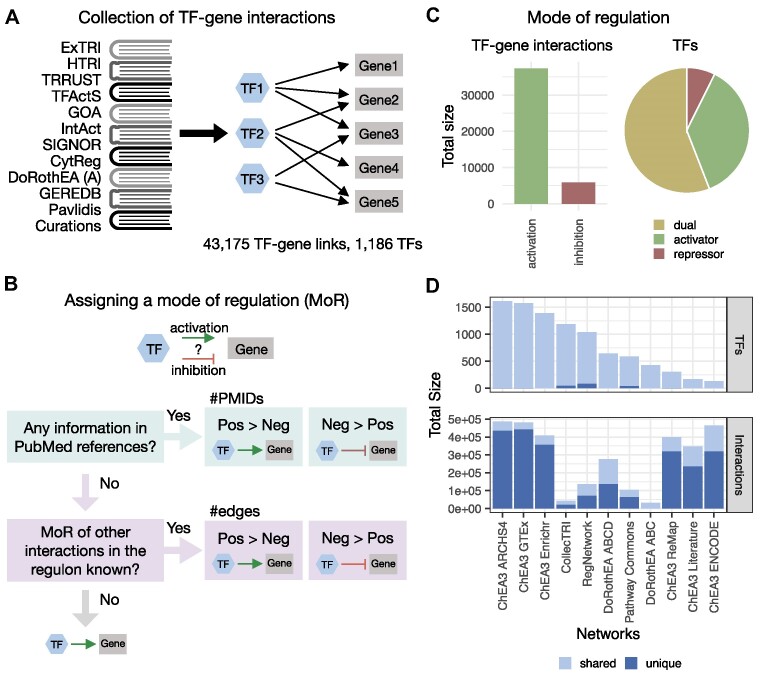
Description of transcription factor (TF)–gene interactions in the CollecTRI-derived regulons and comparison to other regulon collections. (**A**) Collecting transcription factor (TF)–gene links to construct regulons from CollecTRI. Depicting prior knowledge resources used to collect links, which were aggregated within CollecTRI. (**B**) Flow chart describing how the mode of regulation (MoR) was assigned to each TF–gene link. The MoR, indicating the direction of transcriptional regulation from the TF to its target gene, was determined for each TF–gene link, based on factors such as PubMed references (PMIDs) and the MoR of other genes in the regulon. (**C**) Summary of the MoR for TF–gene interactions in CollecTRI. Total number of interactions for activating and repressive TF–gene links (left) and percentage of TFs that purely function as activators, repressors, or have a dual mode of regulation (right). (**D**) Comparison of the number of unique TFs (top) and interactions (bottom) across different resources—with ChEA3 ARCHS4, ChEA3 GTEx and ChEA3 Enrichr being solely based on co-expression or co-occurrence. Any TF or interaction present in more than one resource is considered shared.

From the compiled information in CollecTRI we constructed signed and directed CollecTRI-derived regulons which can be used for the inference of TF activities. Each regulon consists of all target genes of one particular TF as reported in the meta-resource. To ensure reliability of the TF–gene interactions and account for the overlap across resources, we gathered the unique PMIDs for each TF–gene interaction and removed those lacking any reference (Figure 1A). Furthermore, to focus on proteins with a direct regulatory effect on gene expression, we included only TF–gene links from TFs classified as DNA-binding transcription factors (dbTFs), co-regulatory transcription factors (coTFs) or general initiation transcription factors (GTFs) based on criteria from TFclass (60), Lambert *et al.* (61), Lovering *et al.* (6) or gene ontology (GO) annotations (36).

We then assigned a mode of regulation to each TF–gene pair, indicating the sign of transcriptional regulation from the TF to its target gene. Specifically, we determined whether the TF activates or represses the expression of its target gene. Hereby, activation corresponds to an increase in the expression of the target gene, whereas repression corresponds to a decrease in expression. In order to determine the mode of transcriptional regulation for each TF–gene pair in the CollecTRI-derived regulons, we considered integrating different sources of information (Supplementary Figure S2A) (Methods: Assigning the TF–gene mode of regulation). Initially, we examined the impact of incorporating specific knowledge for each TF–target link, which was based on the prevalence of PubMed references associated with a specific mode of regulation. Additionally, we considered including general prior knowledge about the mode of regulation of a TF, obtained from GO terms (36) and Uniprot keywords (38), as well as structural information about the Krüppel associated box (KRAB) domain, known to function as a transcriptional repressor domain (62), and the classification of effector domains of 594 human TFs provided by Soto *et al.* (39). The effector domain, being capable of activating or repressing the expression of a TF’s target genes through multiple mechanisms, offers supplementary information for categorizing TFs as either activators or repressors (56). We assessed the impact of both approaches for assigning a mode of regulation to the TF–gene interactions, both individually and in combination, by evaluating their influence on the performance of the CollecTRI-derived regulons in recapitulating the changes in gene expression caused by the perturbation of a TF (Methods: Benchmark procedure). Our findings revealed that assigning a mode of regulation based on the prevalence of PMIDs yielded better benchmark performance compared to TF regulons with the mode of regulation for all TF–gene rendered activating (adjusted *P*-value < 2.2 × 10^−16^, *t*-value equal to 103 and 69.6 for AUROC and AUPRC, respectively). However, assigning a general mode of regulation for TFs based on prior knowledge led to a decrease in performance (adjusted *P*-value equal to 6.5 × 10^−5^ and 1.5 × 10^−10^, *t*-value equal to 4 and 6.5 for AUROC and AUPRC, respectively) (Supplementary Figure S2B). Next, we considered information from other interactions within a TF’s regulon to make informed decisions on whether a TF is more likely to activate or repress the expression of its target genes and included this information for TF–gene interactions that lacked direct information from the PMIDs. This additional information significantly improved the benchmark performance of the CollecTRI-derived regulons (adjusted *P*-value equal to 2.5 × 10^−13^ and 1.2 × 10^−33^, *t*-value equal to 7.4 and 12.3 for AUROC and AUPRC, respectively) (Supplementary Figure S2C). Lastly, we evaluated the effect of assigning an activating or repressing mode of regulation by default in cases where no information was available from any of the sources and discovered that a default activating mode of regulation outperformed a default repressing mode of regulation (adjusted *P*-value < 2.2 × 10^−16^, *t*-value equal to 337 and 294 for AUROC and AUPRC, respectively) (Supplementary Figure S2D). In the end, the mode of regulation for each TF–gene interaction in the CollecTRI-derived regulons was determined based on the prevalence of PMIDs and the classification of the TF based on the mode of regulation of other interactions in the regulon, with TF–gene interactions lacking available information being assigned an activating mode of regulation (Figure 1B). For 19541 TF–gene interactions the mode of regulation was determined based on the prevalence of PubMed references. Meanwhile, for 1904 interactions, the mode of regulation was assigned by the TF classification based on other interactions in the regulon. For the remaining 21730 TF–gene interaction an activating mode was assigned by default (Supplementary Figure S2E). The final annotation procedure led to 86% activating and 14% repressing TF–gene links. 56% of TFs were represented with a dual role in regulation, meaning the TF was assigned to either activate or repress the expression of its target genes. 37% of the TFs had only activating links, whereas 7% of TFs are only represented by repressing links. (Figure 1C). With that, the CollecTRI-derived regulons in total cover 1186 TFs and 43175 signed TF–gene interactions.

We then compared the coverage of the CollecTRI-derived regulons to other known collections of TF regulons, namely ChEA3 (27), RegNetwork (29), Pathway Commons (28) and DoRothEA (15). ChEA3 contains a collection of gene set libraries generated from TF–gene co-expression, TF–target associations from ChIP-seq experiments, and TF–gene co-occurrence computed from user-submitted lists to the Enrichr tool. RegNetwork is a manually curated database of experimentally observed or predicted transcriptional and post-transcriptional regulatory interactions. Pathway Commons is a resource that compiles information about regulatory networks as well as biological pathways including molecular interactions, signaling pathways, and DNA binding from different databases. Finally, DoRothEA integrates information on gene regulatory interactions with assigned confidence levels from multiple sources, including literature-curated resources, ChIP-seq peaks, motif analysis, as well as inference from gene expression data. Only DoRothEA among the four regulon collections we compared to the CollecTRI-derived regulons also provides signed information about the direction of transcriptional regulation. For a fair comparison between the TF regulons, all collections were filtered to only contain TF–gene interactions from annotated dbTFs, coTFs or GTFs, as described above.

We then compared the TFs and TF–gene links across all collections of TF regulons, and we found that the CollecTRI-derived regulons exhibit the highest TF coverage (1186) besides the ChEA3 gene set libraries ARCHS4 (1612), GTEx (1578) and Enrichr (1393). It is worth noting that these ChEA3 libraries were generated using co-expression or co-occurrence strategies, which are known to produce a higher number of false positive interactions in TF–target association studies (30). The CollecTRI regulons cover 48 TFs not present in any of the other four resources. RegNetwork and Pathway Commons also provide information on 80 and 42 unique TFs, respectively, otherwise 91.3% of all TFs across the analyzed resources are present in at least two of them. In terms of TF–gene interactions, resources mainly collecting information from curated databases, such as RegNetwork, Pathway Commons, DoRothEA and CollecTRI, generally showed a lower number of interactions. As previously mentioned, TF regulons generated using co-expression and co-occurrence strategies, such as some ChEA3 libraries, tend to have a higher number of potential interactions that often include many indirect regulatory relationships. In general, there was a low overlap between the resources we compared, with an average of 63.8% of interactions being unique to each collection of TF regulons and a general low overlap of target genes for shared TFs across networks (mean jaccard index of CollecTRI regulons compared to all networks equal to 0.01) (Figure 1D, Supplementary Figure S3). Overall, the CollecTRI-derived regulons have an extensive coverage of TFs with high-confidence interactions and, in contrast to most other regulon collections, include information about the sign of the transcriptional regulation.

We also integrated the CollecTRI-derived regulons into the OmniPath database (32), for distribution and integration with other databases. CollecTRI is available by the web service at https://omnipathdb.org/, enabling convenient access by the Python, R and CytoScape (63) OmniPath clients, and connecting it directly to downstream methods and integrated software, such as DecoupleR for TF activity inference (33).

### Systematic comparison of TF activity inference from CollecTRI-derived regulons with other regulon collections

We evaluated the quality of the CollecTRI-derived regulons by assessing how well they are able to recapitulate the changes in gene expression caused by the perturbation of a TF in comparison to other existing regulon collections. As previously described, we reasoned that if a TF’s set of targets is reliable, meaning their expression is regulated by the TF, the regulon's collective expression pattern should be a proxy of the TF’s transcriptional activity (15). To test this, we downloaded perturbation data from KnockTF (31), a comprehensive human gene expression profile database from TF knockdowns and knockouts studies. KnockTF contains manually curated RNA-seq and microarray datasets associated with TFs perturbed by different knockdown or knockout techniques across multiple tissues and cell types. For the benchmark, we restrict the datasets to experiments were the TF perturbation is highly likely to have been effective, by only including data from experiments where the expression of a TF was markedly decreased after its knock down or knock out, leading to a total number of 388 perturbation experiments covering 234 unique TFs (Methods: Benchmark data).

**Figure 2.**
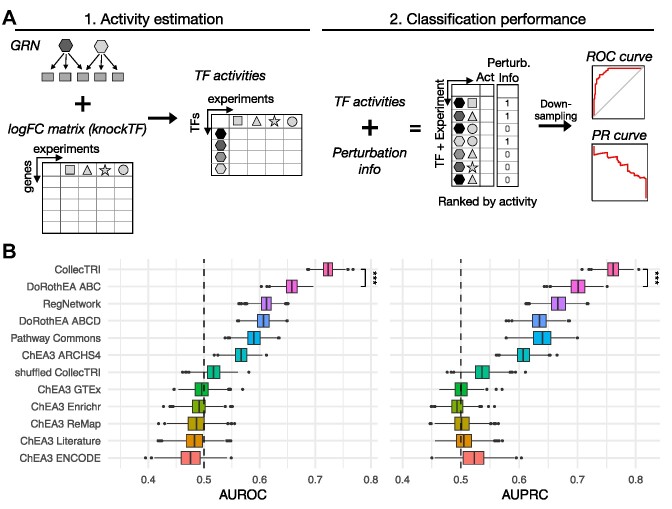
Systematic comparison of collections of transcription factor (TF) regulons. (**A**) Description of benchmark pipeline for the comparison of different regulon collections. First, transcription factor (TF) activities are inferred from the gene expression data of the knockTF perturbation experiments using the regulon information from each resource. TFs are presented as differently colored hexagons and experiments are presented as different shapes. Activities are then aggregated across experiments and ranked by their activity. A downsampling strategy is applied to have an equal number of perturbed and non-perturbed TFs randomly selected 1000 times to calculate area under the Receiver operating characteristic (AUROC) and Precision–Recall curve metrics (AUPRC). (**B**) Predictive performance of TF regulons identifying perturbed TFs in knockTF experiments. AUROC (left) and AUPRC (right) for each regulon collection classifying TFs as perturbed or non-perturbed based on their activities.

We then followed the benchmark pipeline in the decoupler python package (33) to systematically compare the regulons generated from CollecTRI, to the ones from DoRothEA, Pathway Commons, RegNetwork and the ChEA3 libraries. Additionally, we used a permuted version of the CollecTRI-regulons as a baseline of performance. In this version the target genes and mode of regulation in CollecTRI were shuffled and randomly assigned to a TF. As such, these TF regulons do not represent biological information and can thus serve as a baseline of performance. TF activities were then inferred from the differentially expressed genes of each KnockTF experiment using the regulons provided by each resource. Only TF regulons containing at least five target genes among the genes measured in the experiment were used for the activity inference, leading to a restricted number of TFs for each resource (Supplementary Figure S4). All inferred TF activities across experiments were sorted by their activity scores, and the classification of TFs based on the estimated activities compared to the knock-out information was evaluated with the area under the receiver operating characteristic curve (AUROC) and the area under the precision-recall curve (AUPRC) (Figure 2A) (Methods: Benchmark procedure). We performed the comparison of CollecTRI-derived regulons to those from other resources and showed that the CollecTRI regulons had median AUROC and AUPRC values of 0.73 and 0.77, respectively, which were higher than those of all other resources (adjusted p-value < 2.2 × 10^−16^, mean t-value across tests equal to 271.8 and 281.8 for AUROC and AUPRC, respectively) (Supp. File 1, Figure 2B). Furthermore, all ChEA3 libraries, except for ChEA3 ARCHS4, did not exhibit a higher performance compared to the random baseline set by the permuted CollecTRI version (*t*-test: adjusted *P*-value > 0.05). Overall, the results from the benchmark show that the CollecTRI-derived regulons outperform other TF regulon collections in identifying perturbed TFs based on TF activities, suggesting that, of the resources compared, the TF–gene interaction information compiled in CollecTRI provides the most reliable regulons for estimating TF activities.

Since the benchmark data mainly covers TFs that are well studied and usually have a larger number of targets associated with them, we tested if the number of genes regulated by a TF was related to the performance of the networks to predict perturbed TFs. For the top three performing TF regulon collections, we first tested if there was a difference in the number of targets between TFs that were part of the benchmark data set and those TFs that were not. For all three resources, we observed that the TFs included in the benchmark had a higher number of targets associated with them (adjusted *P*-value = 2.61^−5^, 1.34^−3^ and 2.81^−4^, *t*-value = 4.59, 3.27 and 3.84 for CollecTRI, DoRothEA ABC and RegNetwork, respectively) (Supplementary Figure S5A). To assess the relationship between the number of targets and the accuracy estimating TF activities for each experiment included in the benchmark, we computed Pearson correlation coefficients and found that the average correlation across all experiments was equal or <0.4 for all resources, with the CollecTRI-derived regulons showing the lowest mean correlation of 0.19 (Supplementary Figure S5B). Therefore, we concluded that the better performance of the CollecTRI-derived regulons is not influenced by an increased bias towards TFs with a higher number of targets.

Another limitation of the current benchmark is that it disregards possible off-target effects of TF perturbation assuming that the perturbed TF has the most deregulated activity. Thus for a limited collection of 12 TFs where we had multiple perturbation experiments we repeated the benchmark only classifying the activity of the perturbed TFs without including non-perturbed TFs (Methods: Benchmark procedure). In this benchmark setting, we observed a better performance of CollecTRI regulons for the TFs REST, TP53, FLI1, NRF2F2 and SOX2 in comparison to the other networks with average median AUROC and AUPRC value of 0.85 and 0.89, respectively, (adjusted *P*-value < 1.8 × 10^−10^, mean t-value across TFs = 73.4 and 78.8 for AUROC and AUPRC, respectively) and a perfect classification for REST (Supplementary Figure S5A). However, overall all networks performed comparably (Supplementary Figure S6B). Although limited to a few TFs, CollecTRI-derived regulon's performance was comparable to the other networks in this benchmark setting, with an improved performance for specific TFs.

### TF estimation in cancer tissues

**Figure 3.**
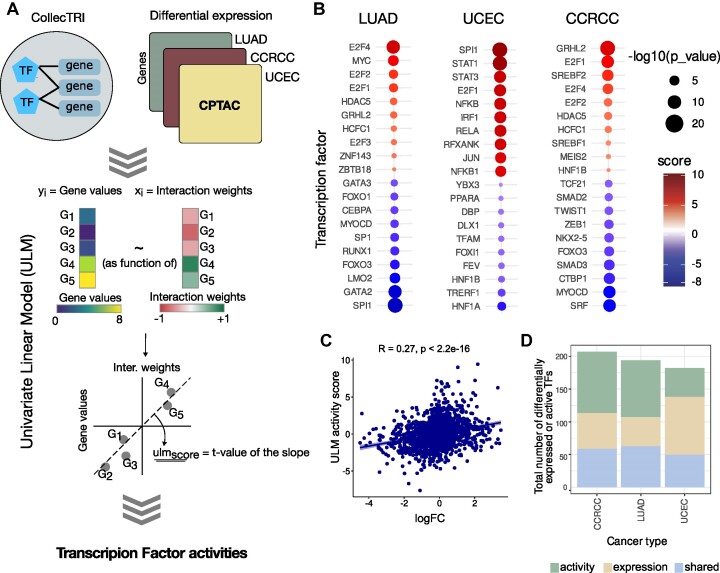
Workflow and results of the case study of transcription factor activity inference using CollecTRI and decoupleR. (**A**) Schematic representation of the workflow for the inference of transcription factor activities from transcriptomics data of Uterine Corpus Endometrial Carcinoma (UCEC), Lung Adenocarcinoma (LUAD) and Clear Cell Renal Cell Carcinoma (CCRCC). (**B**) Transcription factors (TFs) with differential activities as predicted by decoupleR. Each TF is shown as an individual dot and colored based on the inferred activity score. The size of the dot is inversely related to the *P*-value (the bigger the size, the more significant the observation). (**C**) Correlation between predicted activity of a TF (y-axis) and the logarithmic fold change (logFC) compared to normal tissue (x-axis). (**D**) Overview of the number of TFs identified from expression (beige), activity (green) or both (lightblue).

To showcase the value of using CollecTRI-derived regulons for predicting TF activities, we performed a TF activity inference analysis using differential expression data from three cancer types: Uterine Corpus Endometrial Carcinoma (UCEC) (64), Lung Adenocarcinoma (LUAD) (65) and Clear Cell Renal Cell Carcinoma (CCRCC) (66) (Figure 3A). These datasets comprise gene expression data of tumors and adjacent normal tissues from multiple patients.

Based on the differentially expressed transcriptome of tumor versus normal tissue, we predicted TF activities for each cancer type and observed in total 283 significantly deregulated TFs. The activity scores predicted with the univariate linear model (ulm) in decoupler reflect the direction of regulation of a TF (increased or decreased) and its level of activity (high or low) (33). Generally, our analysis reflected previously described TF activity changes in cancer tissue. For instance, proliferation- and cell survival-promoting TFs, such as MYC, Jun-, FOS- and E2F family TFs, were found to have significantly increased activity across the three cancer types. On the other hand, cell death-related TFs, such as members of the FOXO family, were found to have significantly reduced activity (Supp. File 2).

To highlight the added value of the additional TF–gene coverage of CollecTRI, we compared the predictions of TF activities for those TFs whose regulons are included only in CollecTRI-derived regulons and not in DoRothEA ABC, which was chosen as the main network for comparison as it was the second-best performing network in our benchmark. For all three cancer types, approximately 30–40% of the predicted dysregulated TFs were uniquely part of the CollecTRI regulons and not of DoRothEA ABC. Specifically, in UCEC, we identified 94 total dysregulated TFs, out of which 40 TFs were found to be specific to the CollecTRI regulons. Similarly, in CCRCC, we detected 153 dysregulated TFs, with 56 TFs being specific to CollecTRI. Lastly, in LUAD, we predicted 48 CollecTRI-specific TFs out of the total 150 dysregulated TFs. To evaluate the validity and relevance of the ‘CollecTRI-exclusive’ TFs, a literature review for their role in their respective cancer types was conducted, especially focusing on TFs with a high or low activity compared to normal tissue (Figure 3B).

For LUAD, in total five of the 20 TFs predicted to be most dysregulated were uniquely part of CollecTRI regulons, and four of those had a previously reported role in several aspects of LUAD, such as its development and prognosis. HCFC1 is involved in the control of cell cycle and it has been reported to be overexpressed in lung adenocarcinomas (67). Similarly, the upregulation of HDAC5 has been found to promote lung adenocarcinoma by regulating several cell cycle and epithelial-mesenchymal transition genes (68), and in our analysis, CollecTRI regulons predicted its increased activity in LUAD. LMO2 is a tumor suppressor which acts through the regulation of the Wnt pathway in several tumor types. In lung adenocarcinomas and other epithelial-derived tumors, LMO2 was found to have a reduced expression and activity (69), as also predicted in our results. Lastly, MYOCD which is an essential tumor suppressor gene in specific lung cancers (70) was predicted to have a significantly down regulated activity.

Among the top TFs for which we estimated altered activities in UCEC, six were part of only CollecTRI-regulons, four of which had a previously described role in this specific cancer type. SMAD2, has been shown to have tumor-suppressive functions in endometrial carcinoma cells, and the inhibition of its activity has been associated with the constituent activation of the PI3K/AKT pathway, increased proliferation and decreased apoptosis (71). SMAD2 was predicted to exhibit a significantly decreased activity in the UCEC dataset. The aforementioned histone deacetylase, HDAC5, was predicted to have an increased activity with the CollecTRI regulons. HDCA5 inhibition with pan-HDAC inhibitors has been reported to lead to cell cycle arrest in UCEC (72), suggesting a positive role of the TF in cancer development, which is also reflected in the increased predicted activity. The HCFC1 transcription factor has an immunomodulatory role in cancer by inhibiting immune responses, and by promoting tumor growth and vascularization (73). In accordance with its cancer-promoting role, HCFC1 was found to have an increased activity in UCEC. Lastly, the tumor suppressor TCF21 is a hypoxia-driven target of p53 in UCEC (74), and was also predicted to have a reduced activity in our analysis.

CCRCC is one of the three main subtypes of renal cell carcinomas (RCCs), all of which have been described for their distinct transcriptional and epigenetic characteristics. A study by (75) reported the main driving TF of each subtype. Among those main driving TFs, two are found in the predictions by CollecTRI-regulons. ETS1, which was estimated to be overactive by our analysis, was found to be one of the two main TF regulators in CCRCC. On the contrary, FOXI1, a main TF regulator of another RCC, chromophobe RCC, was found to be significantly underactive in CCRCC, as would be expected in this specific subtype. FOXI1 was among the TFs which were exclusively found with the ColleCTRI regulons. TFAM is a mitochondrial TF which is also included in the regulation of pyroptosis. Together with 10 more pyroptosis-related genes, TFAM, was identified as a risk gene for the prognosis of CCRCC (76). Additionally, the YBX3 TF, which was predicted to have a reduced activity in CCRCC, has been suggested to be associated with RCC tumor grading (77). Two additional TFs, TRERF1 and DLX1 were inferred to be more active in tumor than normal tissue. While the role of TRERF1 in CCRCC is poorly understood, TRERF1 is a known regulator of CYP11A1, which is frequently downregulated in CCRCC. DLX1 has no reported role in CCRCC, however, it has a known oncogenic role in other cancer types such as prostate (78) and ovarian (79) cancers.

To assess the relationship between the expression and predicted activity of a TF, we calculated the correlation between the two. As shown in Figure 3C, TF expression and activity displayed only a weak positive correlation. This indicates that alterations in TF expression may not necessarily serve as a reliable indicator of changes in its activity. Furthermore, when examining the number of TFs exhibiting differential expression and/or differential activity, it was observed that in the case of CCRCC and LUAD, approximately double the number of dysregulated TFs were identified based on their activity compared to their expression (Figure 3D). Conversely, in UCEC, there was a contrary trend where more TFs exhibited changes in expression rather than predicted activity.

Overall, this comparative analysis highlights the usefulness of CollecTRI-derived regulons in inferring TF activities. The presence of cancer-type-relevant TFs in the results showcases how the augmented TF coverage in CollecTRI-derived regulons appears to balance the identification of meaningful TFs without overwhelming the output with potentially extraneous information. Additionally, our analysis highlights that TF activity estimation can provide insights on TF dysregulation which cannot be directly drawn only by gene expression changes.

### Transcription factor activity estimation in single-cell RNA-seq

**Figure 4.**
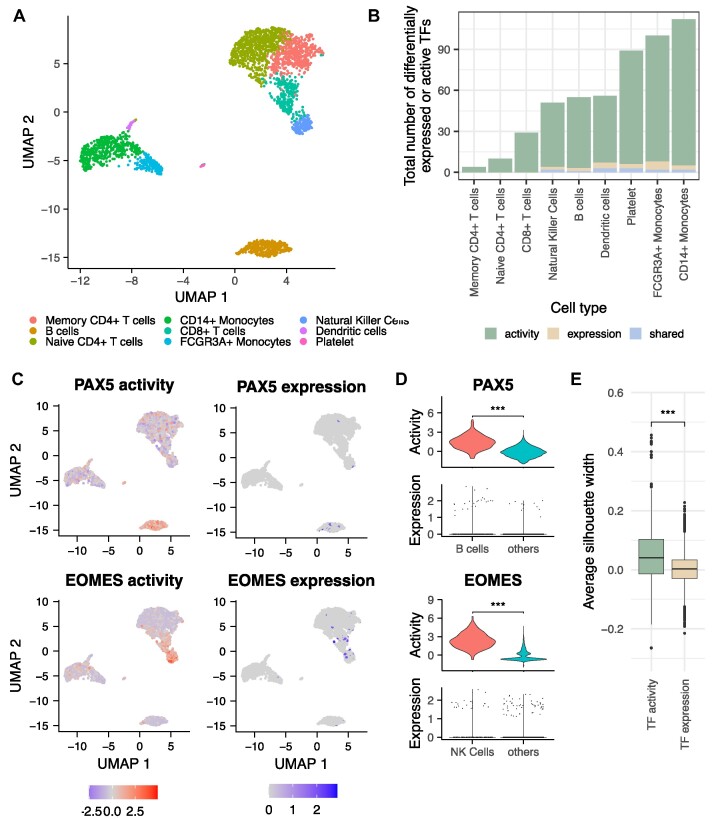
Application of TF activity estimation using CollecTRI on a representative scRNA-seq dataset of peripheral blood mononuclear cells (PBMCs). (**A**) UMAP of scRNA-seq data of PBMCs (*n* = 2638), color corresponds to annotated cell types. (**B**) Overview of the number of marker TFs identified from expression (beige), activity (green) or both (lightblue). (**C**) UMAP of scRNA-seq data of PBMCs showing the activity (top) or expression (bottom) levels of the TFs PAX5 (left) or EOMES (right). TF activities were estimated for each cell using the CollecTRI-derived regulons and the univariate linear model method in decoupler. (**D**) TF activity and expression for PAX5 (top) and EOMES (bottom). The expression and activity of PAX5 and EOMES were compared between the corresponding cell type (B cells for PAX5 and natural killer (NK) cells for EOMES) and all other cell types. For the expression of both TFs, statistical testing was not applied as their expression was captured in less than 15% of cells in the corresponding cell type. (**E**) Comparison of cluster correspondence to annotated cell types from TF activity and TF expression through the average silhouette width of all cells.

We employed the CollecTRI-derived regulons to estimate TF activities in a single-cell RNA-seq dataset of peripheral blood mononuclear cells (PBMCs) to investigate the ability of TF activities in elucidating cell type specific regulatory mechanisms, in particular when compared to TF expression. The single-cell dataset was processed according to the standard preprocessing workflow as described in Seurat (50) and eight different cell types including B cells, CD14+monocytes, FCGR3A+monocytes, naïve CD4+T cells, memory CD4+T cells, CD8+T cells, natural killer (NK) cells, dendritic cells and platelets were identified based on the expression of canonical marker genes (Methods: Single-cell RNA-seq processing) (Figure 4A).

For each cell in the PBMC dataset, we predicted TF activities based on the normalized gene counts using the CollecTRI-derived regulons and identified marker TFs for each cell type once based on their expression and once based on their inferred activity profiles (Supp. Files 3 & 4). From the 506 cell type marker TFs, 93.5% were exclusively identified based on their inferred activities, and 3.9% based solely on TF expression. 2.6% of marker TFs were identified both at the activity and expression level (Figure 4B). Our results suggest that TF activity estimation could elucidate cell type specific regulatory mechanisms that would be missed by looking only at the expression level of TFs. For example, PAX5 is a well-known central regulator in B cell development, controlling their identity and function throughout the process of B lymphopoiesis (80) and EOMES plays a crucial role in NK cell maturation and functionality and its expression is observed across all stages of NK cell development (81,82). However, the expression of both PAX5 and EOMES is only captured in around 6.7% of B and 10.3% of NK cells in the analyzed PBMC dataset, respectively. In contrast to the expression, we observed a consistent high activity of PAX5 and EOMES in their respective cell type across all cells (adjusted *P*-value < 2.2 × 10^−16^, t-value equal to 20.7 and 20.2 for PAX5 and EOMES, respectively) (Figure 4C and D).

To evaluate if the activities of transcription factors are better conserved than their expression within cell types, we compared the potential of the expression and activity of TFs to group cells from the same cell type together, as previously done by Holland *et al.* (83). We calculated a distance matrix between cells using the expression and activity of TFs independently and compared the average silhouette width of all cells using as reference the cell type annotation provided by the atlas. Thereby, a higher silhouette width indicates a better correspondence to the annotated cell types. We observed that using TF activities resulted in higher average silhouette widths than using TF expression (*P*-value < 2.2 × 10^−16^, *t*-value equal to 23.8) (Figure 4E). With that, we concluded that TF activities inferred using the CollecTRI-derived regulons is more informative than TF expression alone and can thus provide new and comprehensive insights into the regulatory mechanism of distinct cell types.

## Discussion

Transcription factor (TF) regulons represent regulatory circuits that depict the coordinated regulation of downstream target genes by TFs. They can be valuable for understanding various biological processes, including development, cell differentiation, tissue homeostasis, and disease progression. To derive functional insights from these regulons, TF activities can be inferred from the expression levels of target genes, as shown in various studies (2,7,11,12). However, to interpret these findings accurately, it is important to critically evaluate the reliability and coverage of TF regulons.

In this paper, we present a well-defined, transparent, and reproducible workflow to generate regulons from CollecTRI, a meta-resource that compiles TF–gene information from 12 different resources including information inferred from text mining, manual curations and a number of publicly available databases (26). With that, the CollecTRI-derived regulons provide the most extensive coverage of TF–gene interactions compared to other collections of regulons that extract TF–gene interaction knowledge solely from literature. Since most publicly available meta-resources of TF–gene interactions contain limited or no information about the mode of regulation of a TF to its target genes, we propose an evidence-driven approach to infer the sign of regulation for each TF–gene link in the CollecTRI regulons, which can also be applied to other comprehensive knowledge bases. We evaluated the approach and confirmed that assigning the sign of regulation to each TF–gene interaction individually based on literature-annotated information leads to more accurate TF activity inference compared to assuming a general activation or repression mode of TFs based on prior knowledge. The limited performance of uniformly assigning a sign to each TF can be explained by TFs often having both activating and repressing effects on gene transcription. The heterogeneity of the regulatory activity of TFs is expected given that gene-specific regulatory promoter and distal elements influence the formation of large protein complexes comprising both DNA-binding TFs (dbTFs) and cofactors (coTFs). Ultimately the composition and state of these protein complexes determine the functional output of transcription regulation (4). Next, through systematic comparison with other known TF regulon collections, we showed that CollecTRI-derived regulons perform best in identifying perturbed TFs based on gene expression, suggesting a high quality in CollecTRI’s TF–gene interactions. Finally, we showcase the value of the CollecTRI regulons by successfully identifying known activity changes in TFs for three different cancer types and identifying cell type marker TFs based on their activities in a single-cell dataset of peripheral blood mononuclear cells (PBMCs).

Despite the good performance of the CollecTRI regulons in the systematic comparison, it is important to bear in mind that the current benchmark is limited to a specific set of TFs. Further perturbation studies would therefore be useful to extend the current benchmark and allow for a more comprehensive evaluation of CollecTRI and other resources.

While the coverage in the CollecTRI regulons is substantially larger than those of other resources, it could still be expanded by including additional TF–gene interactions from other resources, given the low overlap across them. However, identifying high-quality TF–gene interactions within a resource and distinguishing them from indirect regulatory relationships is challenging. Additionally, since CollecTRI is primarily assembled from literature-curated resources, a bias for well-studied TFs may be present, and distinguishing whether a TF has a higher or lower number of target genes as a result of research bias or biological reasons remains challenging.

Another limitation is that the CollecTRI regulons currently only take the sign of regulation into account, omitting the quantitative nature of gene regulation (4). We therefore estimated TF binding weights using motif enrichment analysis, but observed no benefit in the inference of TF activities (Supplementary Note 1, Supplementary Figure S7). Since CollecTRI compiles exclusively TF–gene link interactions omitting cooperative events between TFs and other proteins, distal interactions and the chromatin accessibility landscape among other processes (4), it only captures one layer of the *cis-*regulatory code. This might explain why using TF binding weights did not increase the overall predictability of perturbed TFs.

Finally, the CollecTRI regulons were constructed as generalistic interactions and, as such, do not account for cell type-specific differences nor specific TF-TF cooperativity events (84). Nonetheless, CollecTRI regulons could be used as a building block for context-specific interactions using complementary data types, such as single-cell transcriptomic or chromatin accessibility data. Additionally, the inclusion of TF binding and proteomics data could yield further insights into TF-TF regulatory events (85).

In summary, we constructed a collection of TF regulons with a high coverage of TFs and high confidence TF–gene interactions, which is freely available to the community via the OmniPath (32), DoRothEA (15) and decoupler (33) packages. We conducted a systematic comparison with other known resources, where the CollecTRI regulons showed the best performance in recapitulating changes in gene expression caused by the perturbation of a TF. Additionally, we demonstrated how the regulons can be applied in different biological contexts and can help uncover the role of transcriptional regulation.

## Supplementary Material

gkad841_Supplemental_Files

## Data Availability

The code for the curation of regulatory interactions of CollecTRI and the construction of the CollecTRI-derived regulons is available here: https://github.com/Rbbt-Workflows/ExTRI, https://github.com/saezlab/CollecTRI and on FigShare: https://doi.org/10.6084/m9.figshare. 22362604.v2. Files necessary to reproduce the presented results can be downloaded from https://zenodo.org/record/8192729, including the stationary CollecTRI meta-resource (https://zenodo.org/record/8192729/files/CollecTRI_source.tsv?download=1) and CollecTRI-derived regulons (https://zenodo.org/record/8192729/files/CollecTRI_regulons.csv?download=1). Additionally, the CollecTRI-derived regulons are available in the DoRothEA (https://saezlab.github.io/dorothea/) (15) and decoupler (https://github.com/saezlab/decoupleR) (33) packages through OmniPath (https://omnipathdb.org/) (32), which are also available in Bioconductor (https://bioconductor.org/packages/release/bioc/html/OmnipathR.html, https://www.bioconductor.org/packages/release/bioc/html/decoupleR.html, https://bioconductor.org/packages/release/data/experiment/html/dorothea.html). Please refer to the vignettes on how to access the CollecTRI regulons which are available in R (https://saezlab.github.io/dorothea/articles/dorothea.html#collectri, https://saezlab.github.io/decoupleR/articles/tf_bk.html#collectri-network, https://r.omnipathdb.org/reference/collectri.html) and in python (https://decoupler-py.readthedocs.io/en/latest/notebooks/dorothea.html#CollecTRI-network).
